# Cancer Stem Cell-Exosomes, Unexposed Player in Tumorigenicity

**DOI:** 10.3389/fphar.2020.00384

**Published:** 2020-03-30

**Authors:** Batla S. Al-Sowayan, Alaa T. Al-Shareeda, Bahauddeen M. Alrfaei

**Affiliations:** Stem Cells and Regenerative Medicine Unit, Cell Therapy & Cancer Research Department, King Abdullah International Medical Research Center/King Saud Bin Abdulaziz University for Health Sciences, Ministry of National Guard Health Affairs, Riyadh, Saudi Arabia

**Keywords:** cancer stem cell (CSC), exosome, cancer, cancer cell-derived exosomes, stem cell

## Introduction

Cancer is a well-known, yet poorly understood disease. In which, a healthy tissue is morphed into a cancerous tissue through an intricate, multistep process. This polymorphism has been the focus of cancer research for many decades. Scientists have agreed on a set of traits that are thought to be shared by all cancer tissue types, these traits include; enabling proliferation, evading growth suppressors, resisting cell death, replicative immortality, inducing angiogenesis, and initiating invasion and metastasis, along with other enabling characteristics (Hanahan and Weinberg, 2000; Hanahan and Weinberg, 2011). As researchers investigate the development and propagation of these traits, or as they are called the “hallmarks” of cancer, it became evident that cancer cell-derived extracellular vesicles (EVs), particularly exosomes, play a major role in almost all of them.

In the late 1940s, it was recognized that cells release spherical shaped particles called EVs (Chargaff and West, 1946). Then, almost 40 years later, “exosomes” were acknowledge as a distinct sub-type of EVs (Trams et al., 1981). Up tell now, it is technically challenging to obtain a pure fraction of a specific EV sub-type, due to similarities shared amongst these vesicles. However, the International Society for Extracellular Vesicles, have released a position statement on the minimal experimental requirements for definition of EVs and their functions (MISEV2014; updated in 2018; MISEV2018) (Lotvall et al., 2014; Théry et al., 2018). The MISEV distinction between the different EV sub-types realize on size, density, morphology, subcellular origin, and composition. This was done in order to make scientific reporting on EV biology more consistent and reliable. Most published literature on EVs, including the literature on the role of EVs in cancer, use the term “exosomes” to refer to the EV sub-type under study. These studies include a section that describes the method of “exosome” isolation, and at least a couple of characterization techniques, to justify their nomenclature. Characterization of exosomes in published literature is often based on size and “exosome-enriched” proteins content verification.

On the other hand, the concept of “cancer stem cell” (CSC) only emerged in the 1990s (Lapidot et al., 1994), with a lot of controversy and a number of proposed theories following it. Some say that CSC arise as a result of normal stem cell mutation, while others suggests that CSC arise as a result of a somatic cell acquiring erroneous stem cell characteristics, turning it into cancerous stem cell that can differentiate into heterogeneous population of cancer cells (Baccelli and Trumpp, 2012). Nevertheless, CSCs are now recognized as distinct population of cancer cells, and the CSC-model, is accepted as one of the two most popular models of cancer. The other model being the “clonal evolution-model”, which was described earlier in 1970s. It was postulated that cancer results from the accumulation of mutations in a given group of somatic cell population, within a tissue, thus given raise to heterogeneous population of cancer cells (Nowell, 1976). As the CSC-model becomes more popular, the role of CSCs, as a sub-type of cancer cells, within the tumor microenvironment has recently come to light, especially with advances in stem cell research during the last couple of decades. However, the role of CSC-exosomes, as a sub-type of cancer exosomes, is still under the shadow. Thus, in this article we aim to provide a standpoint on the possible role of CSC-exosomes, and why it should be examined as a separate group of cancer cell-exosomes, based on published literature.

### Exosomes, Devoted Messengers for Good or Bad

Exosomes originate from the inward budding of the early endosomes, which later mature into multivesicular bodies (MVBs) (Doyle and Wang, 2019). Depending on their content, MVBs are either sent to the lysosome to be degraded or released into the extracellular space, forming what’s called exosomes (Doyle and Wang, 2019). Cells of different tissue types were found to release exosomes in order to facilitate intercellular communication, thus initiating different biological actions (Ma et al., 2019). Cancer cells, and cancer-associated cells, within the tumor micro-environment were also found to release exosomes. This allows them to commute their message to malignant and non-malignant cells, and initiate pathways that support tumor survival and propagation (Wortzel et al., 2019). The exosome mediated intercellular communication is enabled through “exosomal cargo”. This includes functional proteins, micro-ribonucleic acid (miRNAs) and messenger RNAs (mRNAs) (Hessvik and Llorente, 2018). Exosomes will deliver its cargo from the releasing cell into the recipient cell, which contains the encrypted message. There is a growing body of published literature on the role of cancer cell-exosomes in promoting cancer progression through enabling recipient cells to acquire the mentioned “hall marks” of cancer. A number of studies, have repeatedly shown that cancer cell-exosomes, of different cancer types, significantly increase cancer cell proliferation and inhibit apoptosis by activating various proposed cellular pathways (Zhang et al., 2018; Qian et al., 2019). Studies have also shown that cancer cell-exosomes stimulate angiogenesis by stimulating endothelial cells viability, migration, and tube formation *via* the transfer of pro‐angiogenic proteins and miRNAs (Yi et al., 2015; Bao et al., 2018; Lin et al., 2018; Yukawa et al., 2018). Likewise, it was reported that cancer cell-exosomes induce replicative immortality *via* the transfer of telomerase reverse transcriptase mRNA from the telomerase activate cancer cell to the telomerase silenced somatic cell (Gutkin et al., 2016). As for metastasis, it is projected that cancer cells induce metastasis by packing its exosomes with promoters of the epithelial–mesenchymal transition (EMT) cascade, to initiate EMT in the neoplastic epithelial cells, within the tumor microenvironment (Webber et al., 2015; Rahman et al., 2016; Xiao et al., 2016). It is also projected that cancer cells will establish a “pre-metastatic” niche through its exosomes. Cancer cells will release its exosomes into the circulation, where they travel to the metastasis site (Costa-Silva et al., 2015; Liu et al., 2016; Syn et al., 2016). There, cancer cell-exosomes will up-regulate the pro-inflammatory molecules, and vascular leakiness, to mobilize cells that constitute the pre-metastatic niche (Costa-Silva et al., 2015; Liu et al., 2016; Syn et al., 2016). Finally, it is projected that while traveling through the circulation, and engraftment into the new tissue, cancer cell-exosomes support cancer cells by allowing them to escape immune surveillance (Mrizak et al., 2015; Muller et al., 2016; Song et al., 2016). Moreover, in addition to the classical hall marks of cancer, it was reported by a recent study that prostate cancer cell-exosomes play a role in transforming local prostate tissue stem cells into CSCs (Ngalame et al., 2018). While another study reported that glioma cell-exosomes induced a “tumor-like” phenotype in bone-marrow mesenchymal stem cells (BMMSCs) (Ma et al., 2019). This was reported to be based on increased proliferation, migration, and invasion rates of treated BMMSCs. In addition to alteration in BMMSCs protein production, including the production of the metastasis-related proteins.

### Cancer Stem Cell, the Black Sheep of the Stem Cell Family

CSCs are cancer cells (found within tumors) that possess characteristics associated with normal stem cells, specifically self-renewal and the ability to differentiate and give rise to different cell types found in a particular cancer specimen i.e. CSCs are tumor-forming cells (Sun et al., 2018). CSCs can be identified by using a set of unified surface markers (i.e. clusters of differentiation (CD); CD44, CD24, CD133), in addition to added tissue specific markers depending on cancer type (Phi et al., 2018). Within the tumor microenvironment, the CSCs are rear and reside in highly specialized niches (Sreepadmanabh and Toley, 2018). The CSCs niche is designed to maintain and protect the CSCs, allowing them to resist many current anticancer treatments (Prieto-Vila et al., 2017). The CSCs niche will also allow the cells to stay dormant for long periods of time, before initiating local recurrent and/or distant metastatic tumors (Plaks et al., 2015). Thus, it is hypothesized that targeting the whole tumor will only slow down tumor expansion while targeting the CSCs, in particular, will jeopardize tumor growth (Garcia-Mayea et al., 2019). At the same time, in regenerative medicine research, it was reported that stem cells and progenitor cells exert their tissue regeneration effects through the release of paracrine factors, mainly exosomes. Studies are consistently showing that injecting the cell-derived exosomes alone, is enough to induce the same regenerative effect as the “whole-cell” transplant approach. For example, it was reported that exosomes derived from embryonic stem cells (Khan et al., 2015), BMMSCs (Zou et al., 2019), and cardiac progenitor cells (Kervadec et al., 2016), all mimic the benefits of injecting their parent cells in a chronic heart failure and myocardial infarction animal models. Thus, it is logical to assume that CSCs function through the same mechanism as other cancer cells and non-cancer stem cells. We can project that CSCs fulfill its “stemness duties” through the release of paracrine factors, with exosomes as a key player.

### What Is Proposed?

As discussed above, cancer cell-exosomes are crucial for tumor initiation, maintenance, and propagation. However, published literature on this subject matter often don’t describe the sub-type of cancer cells that these exosomes were derived from. It is well established by now that cancer cell-exosomes mediate cell to cell communication within the tumor microenvironment, to support and promote tumorigenesis. It is also well established by now that any alteration to parent cell, alters exosome secretion and content, which in turn alters its message. For example, when cancer cells were subjected to hypoxia prior to exosome isolation, to reflect the tumor’s hypoxic environment, these exosomes significantly increased migration and invasion of cancer cells (Li et al., 2016), and tube formation by endothelia cells (Kucharzewska et al., 2013; Hsu et al., 2017), compared with exosomes derived from normoxic cancer cells. Therefore, it could be hypothesized that the sub-population of cancer cells, CSCs, produce exosomes with unique characteristics, and thus functions. Currently, there are only few reports on “CSC-derived exosomes”, and their role in cancer propagation, compared to “non-stem cancer cell-derived exosomes” (**Table 1**). One of the first studies to address this issue reported that the “macrovesicles” that had the *in vitro* and *in vivo* angiogenic effect, in renal cancer, were those driven from the CD105^+^ cancer cell sub-population (Grange et al., 2011). Then later on, one study did a miRNA content comparison, and reported that prostate CSC-derived exosomes have in fact a different miRNA content compared with non-stem prostate cancer cell-derived exosomes (Sánchez et al., 2016). Then, a following study reported that glioma stem cell-derived exosomes promoted angiogenesis by containing a particularly high levels of miRNA-21, which upregulates the vascular endothelial growth factor (VEGF) (Sun et al., 2017). While another study identified 11 miRNAs that are characteristic of gastric CSC-derived exosomes, and suggested that a measurement of these miRNAs in patient serum could be used as a predictor of cancer metastasis (Sun et al., 2017). Other recent CSC-exosomes investigations focusing on their role in metastasis, reported that CSC-derived exosomes promote metastasis by promoting EMT in renal cell carcinoma (Wang et al., 2019) and thyroid cancer (Hardin et al., 2018) *via* the transfer of miRNA-19b-3p and non-coding-RNAs respectively. Whereas other reported on CSC-exosome role in creating a pro-tumoral microenvironment. For example, it was reported that glioblastoma stem cell-derived exosomes direct monocytes toward the immune suppressive “M2” phenotype, through the signal transducer and activator of transcription-3 (STAT3) pathway, creating an immunosuppressive microenvironment (Gabrusiewicz et al., 2018). While colorectal cancer stem cell-derived exosomes promote a pro-tumoral phenotype in neutrophils by increasing interleukin-(IL)-1β expression (Hwang et al., 2019)

**Table 1:** Summary of published work on the distinct role of CSC-derived exosomes in tumorigenicity.

Exosome population	Tumorigenic action	Proposed mechanism of action	Reference
Macrovesicles derived from CD105+ cells of renal carcinoma specimens	Promoted angiogenesis and metastasis both *in vitro* and *in vivo*	miRNA screening showed 24 upregulated, and 33 downregulated miRNAs in CD105^+^ macrovesicles compared to CD105^−^ macrovesicles. This distinct miRNA composition favor tumor growth and invasion.	(Grange et al., 2011)
			
Exosomes derived from CD133+ cells of glioblastoma cell line	Increased the *in vitro* angiogenic capacity of endothelial cells	miRNA analysis revealed elevated levels of miRNA-21 in the CD133+ cells, hypothesizing that the derived exosomes promoted angiogenesis through the miRNA-21/VEGF pathway.	(Sun et al., 2017)
			
Exosomes derived from CD105+ cells of clear cell renal cell carcinoma specimens	Induced EMT of cancer cells *in vitro*, and promoted metastasis *in vivo*	miRNA analysis revealed elevated levels of miRNA-19b-3p in the CD105+ cell-derived exosomes. This in turn affected the protein levels of PTEN, a key mediator of cell migration.	(Wang et al., 2019)
			
Exosomes derived from spheroid formations of thyroid cancer cell lines	Induced *in vitro* EMT in normal and non-cancerous thyroid cells	miRNA analysis revealed elevated levels of MALTA1, EMT marker SLUG and stem cell marker SOX2, in exosome treated cells.	(Hardin et al., 2018)
			
Exosomes derived from glioblastoma cell lines cells cultured in stem cell-permissive medium	Polarized monocytes into M2 macrophage phenotype	Western Blot analysis revealed up regulation of PD-L1 in exosome-treated monocytes. PD-L1 correlates with increased STAT3 pathway phosphorylation, which mediate this immune suppressive switch.	(Gabrusiewicz et al., 2018)
			
Exosomes derived from spheroid formations of colorectal cancer cell line	Prompted a pro-tumoral phenotype in neutrophils	miRNA and ELISA analysis revealed elevated levels of IL-1β in exosome-treated neutrophils and their condition medium.	(Hwang et al., 2019)

PTEN, phosphatase and tensin homolog; MALTA1, metastasis associated lung adenocarcinoma transcript 1; PD-l1, programmed death-ligand 1; ELISA, enzyme-linked immunosorbent assay.

Since tumor–host cross-talk is believed to be initiated by CSCs, and communication between cancer cells and other cells is conducted through exosomes, it’s of great importance to take a closer look at the role of CSCs-exosomes, and its involvement in tumor aggressiveness. Also, to examine their miRNA content, compared to non-stem cancer cell- exosomes, in order to postulate mechanisms of actions. Then finally, develop a cancer management strategy that targets CSCs, and involves blockage of the CSC-exosome release channels.

## Discussion

CSCs generate tumors through the stem cell processes of self-renewal and differentiation into multiple malignant cell types. Based on advances in cell signaling biology, it’s expected that these CSCs function through its exosomes. The term “exosome” was used in this article due to the fact that published literature describing EVs role in cancer often refer to the EV sub-type being examined as exosomes. These publications offer reasonable evidence that the EV sub-type being examined is in fact exosomes, *via* various methods of characterization. Other sub-types of EVs i.e. ectosomes, microvesicle particles, and apoptotic bodies, could be released by cancer cells/CSCs, and could play a role as well. However there is no adequate reporting on this in the literature. Therefore, based on findings on the role of cancer cell- exosomes, and the role of CSCs in cancer, the role of “CSC-exosomes” should be investigated as a separate entity. Such studies will encounter a significant technical and quality control issues related to harvestation of a pure CSC population, and subsequent yield of pure CSC-exosome fraction. Nevertheless, the knowledge provided by these studies will be crucial in developing a more effective approaches to control progression and metastasis of tumors and prevent recurrence.

## Author Contributions

BA-S conceptualized and wrote the article. Other authors were involved in manuscript review and editing.

## Conflict of Interest

The authors declare that the research was conducted in the absence of any commercial or financial relationships that could be construed as a potential conflict of interest.

## Abbreviations

EV, Extracellular Vesicle; CSC, Cancer Stem Cell; MVBs, Multivesicular Bodies; miRNA, micro Ribonucleic Acid; mRNA, messenger Ribonucleic Acid; EMT, Epithelial–Mesenchymal Transition; BMMSC, Bone-Marrow Mesenchymal Stem Cell; CD, Clusters of Differentiation; VEGF, Vascular Endothelial Growth Factor; STAT3; Signal Transducer and Activator of Transcription-3; IL, Interleukin.
